# Structural and Functional Insights into Viral and Fungal Proteins Involved in Chronic Inflammation and Their Biologic Treatments

**DOI:** 10.3390/pharmaceutics17111466

**Published:** 2025-11-13

**Authors:** Mohamed Halawa, Alicia L. Gallo, Valerie J. Carabetta

**Affiliations:** Department of Biomedical Sciences, Cooper Medical School, Rowan University, Camden, NJ 08103, USA; halawa@rowan.edu (M.H.), aliciagallo@mail.rossmed.edu (A.L.G.)

**Keywords:** chronic inflammation, antiviral peptides, antifungal peptides, NF-κB pathway, biologics, conjugated therapeutic proteins, monoclonal antibodies, mAbs

## Abstract

Chronic inflammation constitutes a significant characteristic of sustained infections caused by viral and fungal pathogens, with a strong correlation to the development of cancer, autoimmune disorders, and tissue fibrosis. Viral proteins such as HIV-1 Tat, HBV X (HBx), HPV E6/E7, and EBV LMP1 modulate the host’s immune signaling pathways, primarily through the activation of the NF-κB signaling cascade and the disruption of cytokine equilibrium. These molecular interactions result in a pro-inflammatory microenvironment that facilitates viral persistence, immune evasion, and the process of oncogenesis. Structural investigations have elucidated the mechanisms by which these viral proteins interact with host signaling complexes, thereby highlighting their potential as viable therapeutic targets. Similarly, fungal proteins, including secreted aspartyl proteases (Saps), ribotoxin Asp f1, and chitin-binding proteins, incite chronic inflammation by activating pattern recognition receptors and triggering inflammasome activation. Despite the limited structural information of these fungal proteins, emerging models and bioinformatic analyses identified conserved motifs that are crucial for host interactions. Biologic therapies, encompassing antiviral and antifungal peptides as well as monoclonal antibodies, are currently under development to disrupt these protein-host interactions and modulate inflammatory responses. This review provides structural and functional insight into viral and fungal inflammatory proteins and evaluates the potential of biologics as targeted therapeutic interventions for chronic inflammation associated with infections. We discuss the ongoing clinical trials involving neutralizing antibodies targeting HIV, peptide vaccines aimed at HPV and other promising molecules. Finally, we discuss the current limitations of biologics and possible solutions to translate these promising therapeutics into clinical practice.

## 1. Introduction

Chronic inflammation represents a sustained and dysregulated immune response that is involved in the pathogenesis of a myriad of human diseases, encompassing cancer, autoimmune disorders, and organ fibrosis [1]. Persistent infections of viral and fungal agents are significant contributors to this inflammatory condition. In such instances, microbial proteins avoid immune clearance mechanisms and actively interact with or reprogram host immune signaling pathways, resulting in increased cytokine production, immune cell recruitment, and tissue remodeling [2]. An in-depth understanding of the molecular mechanisms through which viral and fungal proteins perpetuate chronic inflammation is crucial for the advancement of targeted immunotherapies and biologics.

In the context of viral infections, proteins synthesized by HIV-1, hepatitis B virus (HBV), human papillomavirus (HPV), and Epstein–Barr virus (EBV) have been rigorously investigated for their immunomodulatory functions. Examples include the HIV-1 trans-activator of transcription (Tat), HBV X protein (HBx), HPV E6 and E7, and EBV latent membrane protein 1 (LMP1, [3,4,5]). Despite their structural and functional heterogeneity, these proteins exhibit a shared inflammatory mechanism; the activation of the nuclear factor-kappa B (NF-κB) pathway and the subsequent dysregulation of cytokine signaling. The NF-κB pathway functions as a regulator of immune and inflammatory responses, via transcriptional activation of genes involved in cytokine synthesis, cellular proliferation, and survival. The canonical NF-κB pathway is the most extensively studied mechanism of activation. It is induced by pro-inflammatory cytokines, such as tumor necrosis factor-α (TNF-α) and interleukin-1β (IL-1β), pathogen-associated molecules including lipopolysaccharide (LPS), or cellular stress signals [6]. In quiescent cells, NF-κB dimers are sequestered in the cytoplasm by inhibitory IκB proteins. Activation occurs by engagement with cell surface receptors that recruit adaptor proteins and activate the IκB kinase (IKK) complex [7]. The IKK complex subsequently phosphorylates IκB proteins, marking them for ubiquitin-mediated degradation by the proteasome. This process liberates NF-κB dimers, which then translocate to the nucleus, bind to κB DNA motifs, and initiate transcription. NF-κB-regulated genes include those encoding pro-inflammatory cytokines (TNF-α, IL-6, IL-1β), chemokines, adhesion molecules, and anti-apoptotic proteins, which enhances inflammation and promoting cellular survival. The canonical pathway is distinguished by its rapid yet transient activation, ensuring prompt responses to infectious agents or tissue damage [8]. Conversely, the non-canonical NF-κB pathway operates at a slower pace, is more stringently regulated, and is activated by a more limited array of stimuli. It is predominantly induced by members of the tumor necrosis factor receptor (TNFR) superfamily, which play critical roles in adaptive immunity, lymphoid organogenesis, and B-cell functionality. Unlike the canonical pathway, the activation of the non-canonical route does not depend on the IKK complex [9].

The dysregulation of the NF-κB pathway contributes to chronic inflammation and fosters the development of a tumor-promoting microenvironment [10]. This chronic inflammatory milieu also promotes viral persistence and immune dysfunction. In response to these challenges, a variety of biologic therapies have emerged, including antiviral peptides that inhibit viral entry or replication, and monoclonal antibodies (mAbs) that target viral envelope proteins or modulate immune responses in the context of chronic viral infections [11]. For example, broadly neutralizing antibodies against HIV-1 are currently undergoing clinical trials, including vrc01 and 3BNC117 [12,13], while therapeutic antibodies such as nivolumab and pembrolizumab are being investigated for their efficacy in virus-associated cancers, including HPV-associated cervical cancer, EBV-associated nasopharyngeal carcinoma, and HBV- or HCV-related hepatocellular carcinoma [14].

Fungal infections, particularly among immunocompromised populations, represent significant contributors to chronic inflammation. Pathogens such as *Candida albicans*, *Aspergillus fumigatus*, and *Cryptococcus neoformans* secrete numerous proteins that continuously engage the host immune response [15]. In contrast to viral proteins, fungal inflammatory proteins predominantly function through extracellular mechanisms, by interacting with pattern recognition receptors (PRRs) such as Dectin-1 and Toll-like receptors (TLRs), activating inflammasomes, and modulating the activities of immune cells, including macrophages and neutrophils [16]. These interactions result in excessive activation of the innate immune system and prolonged inflammatory responses. Notable fungal proteins include secreted aspartyl proteases (Saps), mannoproteins, and chitin-binding proteins [17]. Although the structural elucidation of fungal inflammatory proteins remains limited, advancements have been made in the development of antifungal peptides that disrupt fungal membranes or biofilm formation, and mAbs directed against fungal antigens, such as *Candida* adhesins or *Aspergillus* allergens. These biologics hold promise not only for achieving fungal clearance but also for the modulation of inflammation associated with persistent fungal infections [18].

In this review, we discuss the structural and functional attributes of viral and fungal proteins implicated in chronic inflammation, concentrating on two distinct mechanistic criteria: the activation of NF-κB and cytokine dysregulation for viral proteins, and PRR engagement, inflammasome activation, and immune cell modulation for fungal proteins. Furthermore, we explore the current landscape of antiviral and antifungal biologics, including peptides and mAbs that have been developed or are under investigation for their ability to target these pathogens and their associated inflammatory consequences. We will highlight both the analogous and distinct methodologies employed by viruses and fungi in the maintenance of inflammatory responses and provide guidance for the advancement of targeted immunotherapies for chronic inflammatory diseases associated with infections.

## 2. Viral Proteins Associated with Chronic Inflammation: Structural and Functional Analyses

**Figure 1:**
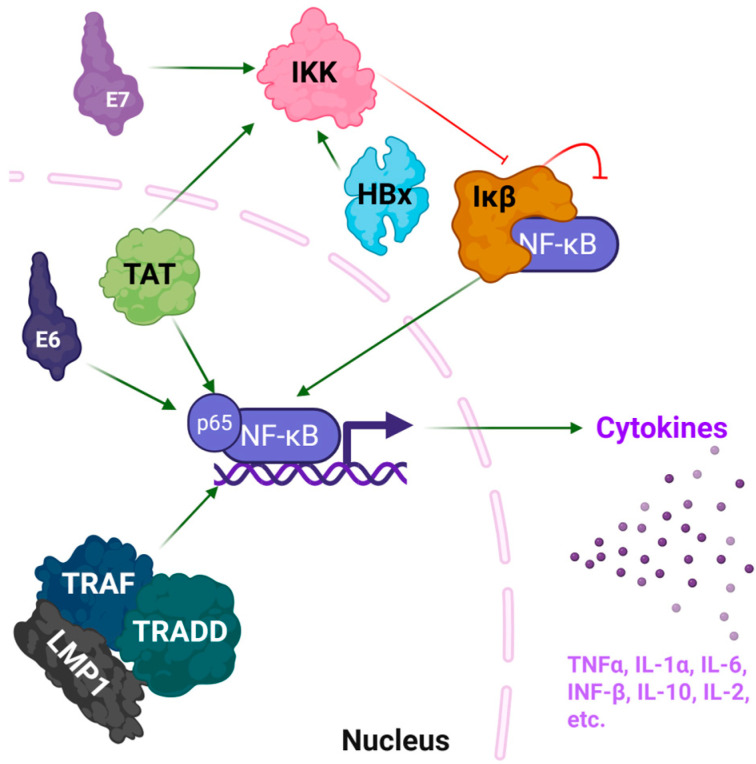
Summary of viral proteins and their mechanism of inflammation. The HIV-1 transactivator of transcription (TAT) and HPV E6 proteins interact with the p65 subunit of nuclear factor-kappa B (NF-κB), leading to direct activation. Similarly, the EBV latent membrane protein 1 (LMP1) mimics the tumor necrosis factor receptor, interacts with the adaptor proteins TRAF and TRADD, and leads to overstimulation of NF-κB signaling. HPV E7, HBV HBx, and Tat interact with the IκB kinase (IKK) complex, leading to degradation of Ikβ and the transport of NF-κB into the nucleus, where it activated target genes. NF-κB activation leads to production of pro-inflammatory cytokines, including TNFα, IL-6, and IL-10. Created in BioRender. Carabetta, V. (2025) https://BioRender.com/rrbgicu.

Chronic viral infections are associated with prolonged immune activation and inflammation, frequently driven by specific viral proteins that disrupt host signaling pathways. Proteins derived from HIV-1, HBV, HPV, and EBV manipulate the NF-κB signaling cascade and cytokine networks, prompting a sustained pro-inflammatory microenvironment that fosters viral persistence, immune evasion, and, in numerous instances, tumorigenesis (Figure 1, [19]). Next, we discuss these viral proteins that contribute to chronic inflammation, emphasizing their structural characteristics and available three-dimensional configurations.

### 2.1. HIV-1 Tat

**Figure 2:**
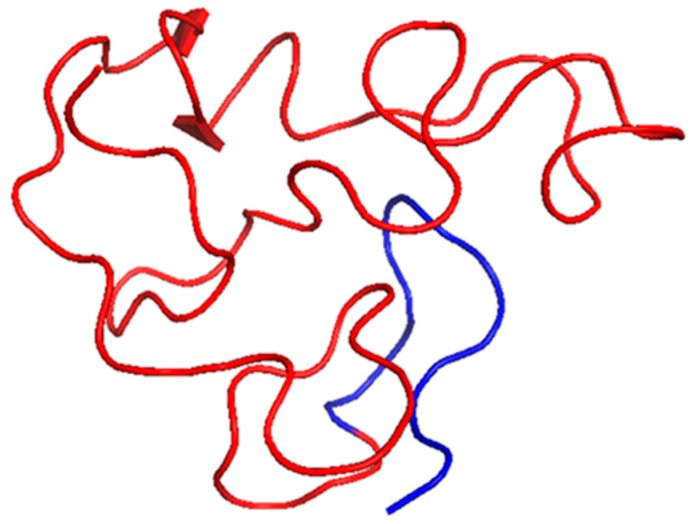
Tat (residues 1–72) structure from subtype B (Red, PDB: 1JFW), with the C terminus represented in blue. This structure was visualized using PyMOL software, version 2.5.8. This structure shows a flexible, elongated conformation with transient α-helical regions.

The HIV-1 Tat protein is necessary for viral transcription and concurrently functions as a secreted pro-inflammatory effector. Tat binds to p65 (RelA) of the NF- κB to activate signaling and enhance the expression of the proinflammatory cytokines (Figure 1). It also activates the IKK complex, leading to increased translocation into the nucleus [20]. From a structural perspective, Tat is intrinsically disordered but encompasses segments with defined secondary structures that promote interactions with host proteins and nucleic acids [21]. The NMR solution structure of a truncated form of Tat (residues 1–72) from subtype B is available (Table 1, Figure 2). Tat effectively crosses cell membranes and induces inflammatory responses in uninfected cells, which contributes to HIV-associated systemic and neuroinflammation. Tat also effects endothelial cells and astrocytes, which leads to HIV-associated neurocognitive disorders, and represent a defining feature of chronic inflammation during HIV infection [22].

### 2.2. HBV X Protein

**Figure 3:**
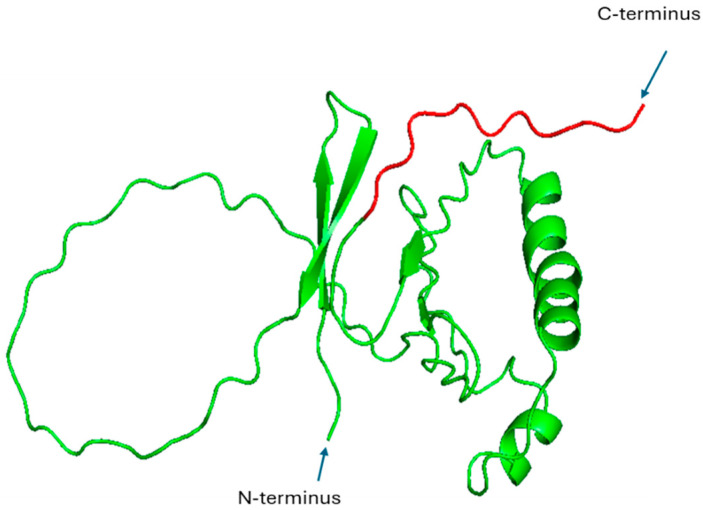
The predicted structure of HBx was generated using AlphaFold and visualized in PyMOL, version 2.5.8. The red coloration represents the C-terminal region, which has transactivation function and is likely responsible for transcriptional activation of both viral and host genes.

HBx is a multifunctional protein that coordinates HBV replication and modulates the signaling pathways of host cells. It activates NF-κB through direct interactions with the IKK complex and the inhibition of IκBα, culminating in the nuclear translocation of NF-κB and the subsequent expression of inflammatory genes (Figure 1). Additionally, HBx plays a role in the survival of hepatocytes, the induction of oxidative stress, and tumorigenesis within the context of chronic HBV infection [28]. Structurally, the characterization of HBx is challenging due to its partially disordered nature and propensity for aggregation. As no complete crystal or NMR structure currently exists in the Protein Data Bank (PDB), we used AlphaFold to predict its structure. This prediction shows that HBx comprises multiple helical segments and a flexible C-terminal region, which could be important for transcriptional activation by HBX of both viral and host genes (Table 1, Figure 3, [29]).

### 2.3. HPV E6 and E7 Proteins

**Figure 4:**
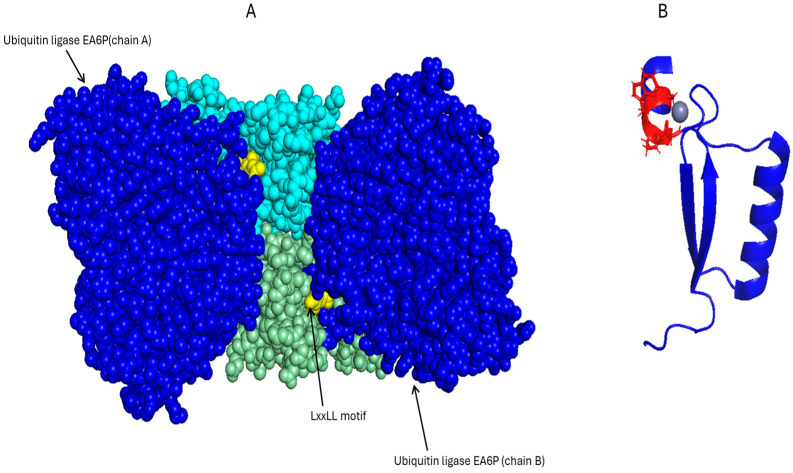
(**A**) The HPV16 E6 structure (PDB: 4GIZ) forms a dimer, with each monomer bound to the ubiquitin ligase (blue) E6AP LxxLL motif (yellow spheres). To show the dimeric arrangement, rather than the N- and C-terminal domains of a single E6 molecule, the two E6 monomers are colored cyan and pale green. (**B**) The CxxC motif in HPV E7′s C-terminal zinc-binding domain is highlighted in red (PDB: 2EWL). The grey sphere represents a coordinated zinc ion. PyMOL, version 2.5.8 was used to visualize all structures.

The oncogenic proteins E6 and E7 of HPV are important for the viral life cycle and the underlying pathogenesis of HPV-related malignancies. E6 facilitates the degradation of p53, while E7 inactivates the retinoblastoma (Rb) tumor suppressor protein, resulting in unregulated cellular proliferation. Furthermore, both proteins have been associated with the promotion of chronic inflammation through the activation of NF-κB and the upregulation of cytokines (Figure 1, [30,31]). E6 directly influences the binding of NF-κB to promoter regions [32], while E7 activates the IKK complex, leading to Iκβ degradation and NF-κB translocation to the nucleus [33]. The structure of HPV16 E6 in association with the LxxLL motif of the E6AP ubiquitin ligase is available (Table 1, Figure 4A). This crystallographic analysis revealed the presence of two zinc-binding domains that facilitate substrate recognition and interaction with host proteins. These domains provide the mechanism of specificity toward the LxxLL motif and stability during binding to the E6AP ubiquitin ligase, which targets p53 for proteolytic degradation [34]. E6 comprises an N-terminal CR1/CR2 domain that is responsible for binding to Rb, which regulates the cell cycle, and a C-terminal zinc-binding domain (Figure 4A, [25]). There is no comprehensive, full-length structure of HPV E7, but there is a structure of the C-terminal domain (Figure 4B). The C-terminal domain contains a conserved zinc-binding motif (CxxC), where C represents cysteine residues and x represents any amino acid. This motif coordinates a zinc ion, which is needed to stabilize the three-dimensional structure, and promote interactions between proteins [35]. This structural integrity allows E7 to disrupt numerous cellular pathways, including those governing cell proliferation and immune response mechanisms [36]. A comprehensive understanding of these structural foundations not only clarifies their contributions to disease progression but also guides the development of targeted therapeutic strategies [26].

### 2.4. EBV LMP1

**Figure 5:**
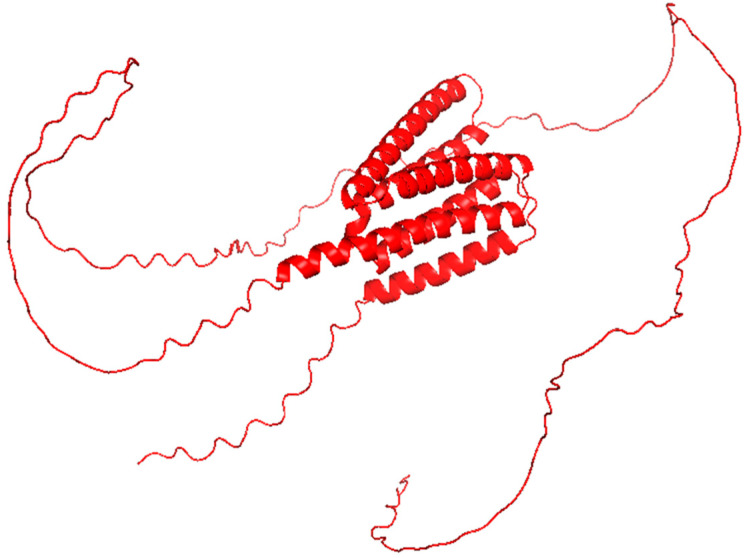
The predicted structure of LMP1 generated using AlphaFold and visualized in PyMOL, version 2.5.8.

LMP1 is a transmembrane protein that operates as a constitutively active analogue of the TNFR. LMP1 interacts with the adaptor proteins TNF receptor-associated factor (TRAF) and TNFR1-associated death domain (TRADD) protein via its cytoplasmic C-terminal activation regions (CTAR1 and CTAR2), thereby connecting receptors to intracellular signaling pathways [37]. This interaction facilitates the activation of both canonical and non-canonical NF-κB signaling pathways, resulting in the upregulation of pro-inflammatory cytokines IL-6 and IL-8 (Figure 1). This results in sustained B cell activation, immune evasion, and chronic inflammatory responses, each of which contributes to viral persistence and oncogenesis [27,38]. LMP1 is associated with EBV-induced malignancies, including Hodgkin’s lymphoma and nasopharyngeal carcinoma, wherein inflammation leads to disease progression [39]. For LMP1, due to its multiple transmembrane domains and high flexibility, the full-length structure remains unresolved. We predicted a structure with AlphaFold (Table 1, Figure 5). The hypothesized structure revealed a configuration comprising six transmembrane α-helices integrated within the membrane domain, interconnected by long, unstructured intracellular and extracellular loops. Of particular interest, CTAR1 and CTAR2 protrude into the cytosol and are represented as partially disordered domains, which likely facilitates the recruitment of TRAF and TRADD.

## 3. Fungal Proteins Involved in Chronic Inflammation: Structural and Functional Insights

**Figure 6:**
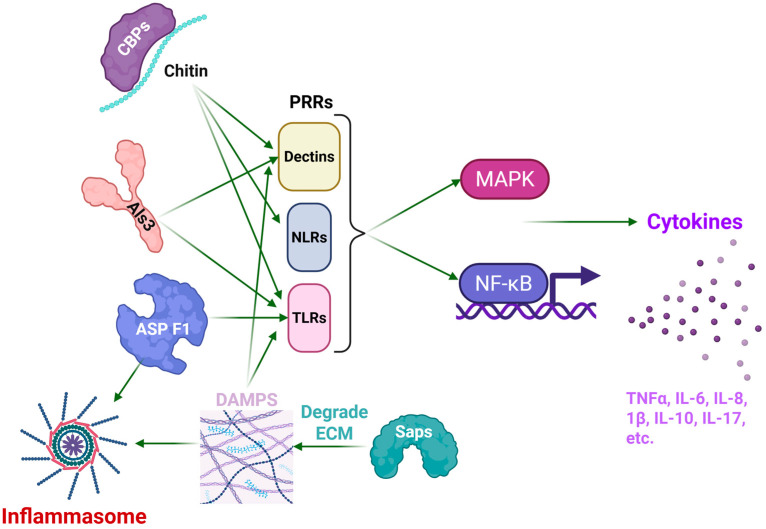
Summary of fungal proteins or constituents that lead to inflammation. The *A. fumigatus* protein AspF1 is recognized by toll-like receptors (TLRs), while the cell-wall component chitin is recognized by TLRs, Dectins and Nod-like receptors (NLRs). Fungal secreted chitin binding proteins (CBPs) protect the chitin from immune cell detection. The *C. albicans* protein Als3 is recognized by Dectins and TLRs. The *C. albicans* Saps lead to degradation of the extracellular matrix (ECM), which releases collagen, laminin, and fibronectin, all of which can serve as damage-associated molecular patterns (DAMPs) and activate TLRs and Dectin-1. Asp F1 and DAMPs also activate the NLRP3 inflammasome. Following activation of the pattern recognition receptors (PRRs), the downstream effects lead to activation of the MAPK and NF-κB signaling pathways, which leads to extensive cytokine production and inflammation. Created in BioRender. Carabetta, V. (2025) https://BioRender.com/nrz7c5k.

Fungal pathogens, such as *C. albicans*, *A. fumigatus*, and *C. neoformans*, are increasingly recognized as significant contributors to chronic inflammation, particularly in immunocompromised individuals [15,40]. Unlike viral proteins, which primarily manipulate intracellular host signaling pathways, fungal proteins exert their effects through interactions with extracellular and membrane-associated immune receptors. The shared mechanisms through which fungal proteins induce chronic inflammation involve the engagement of PRRs, including Dectin-1, TLRs, and C-type lectins, the activation of inflammasome complexes, particularly the NLRP3 inflammasome, and the modulation of immune cell functions, such as macrophage polarization and neutrophil recruitment (Figure 6 [41,42]). In the subsequent sections, we discuss several fungal proteins that are recognized for driving persistent inflammation, with a particular focus on their structural attributes and their implications in shaping the host immune response.

### 3.1. Saps from C. Albicans

**Figure 7:**
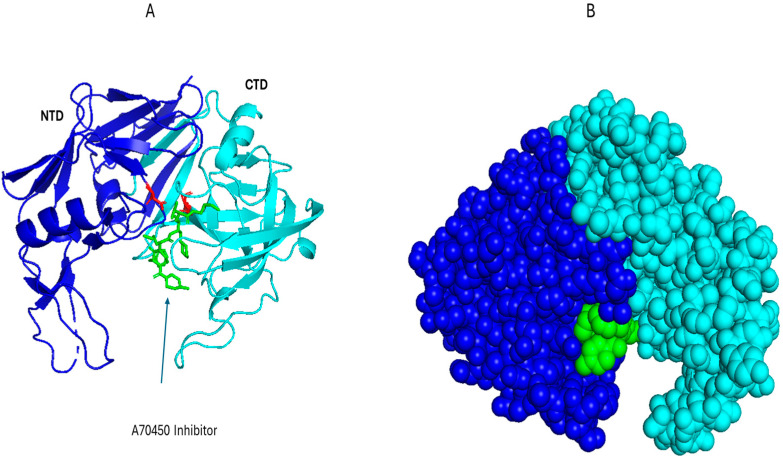
Structural insights into Sap2. (**A**) The structure of Sap2 domains as a cartoon representation, showing the N-terminal domain (NTD) in blue and C-terminal domain (CTD) in cyan, with the A70450 inhibitor depicted in green and the conserved catalytic dyad (Asp32 and Asp218) in red (PDB: 1EAG), visualized in PyMOL, version 2.5.8. (**B**) The structure of Sap2 using spheres to illustrate how the NTD and CTD interact to form the active-site cleft. The colors are depicted as in panel A.

Saps represent a family of hydrolytic enzymes secreted by *C. albicans* that facilitate the degradation of host proteins to promote tissue invasion, including extracellular matrix (ECM) components such as collagen, laminin, and fibronectin. Among the various isoforms, Sap2 is the most investigated variant, which is involved in fungal pathogenesis and the modulation of host immune responses. The molecular architecture of Sap2 has been solved by X-ray crystallography at a resolution of 2.1 Å, which revealed a characteristic bilobed conformation typical of aspartic proteases. Sap2 contains distinct N- and C-terminal domains that have a substrate-binding cleft, with a well-conserved catalytic dyad (Asp32 and Asp218, Table 2, Figure 7A,B [43]). Such an arrangement gives it specificity and operational efficacy under acidic environments, akin to those found at mucosal surfaces and within phago-lysosomal compartments [44,45,46]. The enzymatic catabolism of ECM proteins by Sap2 not only facilitates fungal invasion into host tissues but also leads to the liberation of damage-associated molecular patterns (DAMPs). These DAMPs are recognized by PRRs including TLR4, Dectin-1, and various C-type lectin receptors (Figure 6). Moreover, the cellular stress induced by Sap2 and the degradation of host proteins leads to the activation of the NLRP3 inflammasome, a cytosolic complex that is important for the maturation and secretion of pro-inflammatory cytokines such as IL-1β and IL-18 [47,48]. Sap2 also results in the polarization of macrophages and augmentation of the recruitment of neutrophils, both of which enhance the mucosal inflammatory responses [49].

The small-molecule inhibitor A-70450 binds Sap2 with high affinity and occupies the active-site cleft, which emulates the transition state during peptide cleavage [50]. Extended exposure to Saps, particularly Sap2, is associated with chronic mucosal inflammation, as evidenced in conditions such as recurrent vulvovaginal candidiasis, where sustained immune activation and epithelial damage contribute significantly to the persistence and severity of the disease [44]. While A-70450 is potent in vitro, there was no protective effect in murine disseminated candidiasis models [51,52]. This could be due to the expression of additional, different Sap isoforms during system infection, so inhibition of Sap2 alone would be insufficient, or poor tissue distribution of the peptide. These discouraging early fundings likely contributed to the lack of further development of A-70450. However, with proper investigation, A-70450 could serve as the foundation for potent antifungal agents.

**Table 2:** Summary of fungal proteins that induce inflammation.

Fungal Protein	Species	Mechanism of Inflammation	Structural Features	PDB Code	Binding Motif or Interface	Ref.
Sap2	*C. albicans*	TLR4, Dectin-1; NLRP3 inflammasome activation; cytokine induction (IL-1β, IL-6, TNF-α)	Pepsin-like fold; active-site cleft	1EAG	Active-site Asp residues + substrate-binding loops mediate TLR engagement	[53]
Asp f1	*A. fumigatus*	TLR2/TLR4 activation; Th2 response; eosinophilic inflammation	Conserved α/β ribotoxin topology	1JBS	Ribotoxin loop regions interact with TLR extracellular domains	[54]
Chitin	Multiple	Dectin-1, TLR9, NOD receptor engagement; IL-17 production; granuloma formation	β-sandwich fold (modelled from bacterial CBP21)	2BEM	Glucan-binding surface recognized by Dectin-1 CRD	[55]
Als3	*C. albicans*	Binds E-cadherin and N-cadherin, triggers NF-κB and MAPK signaling	β barrel of the N-terminus	4LE8	β barrel of the N-terminus binds to the host cadherin receptors.	[56]

### 3.2. Asp f1 of A. fumigatus

**Figure 8:**
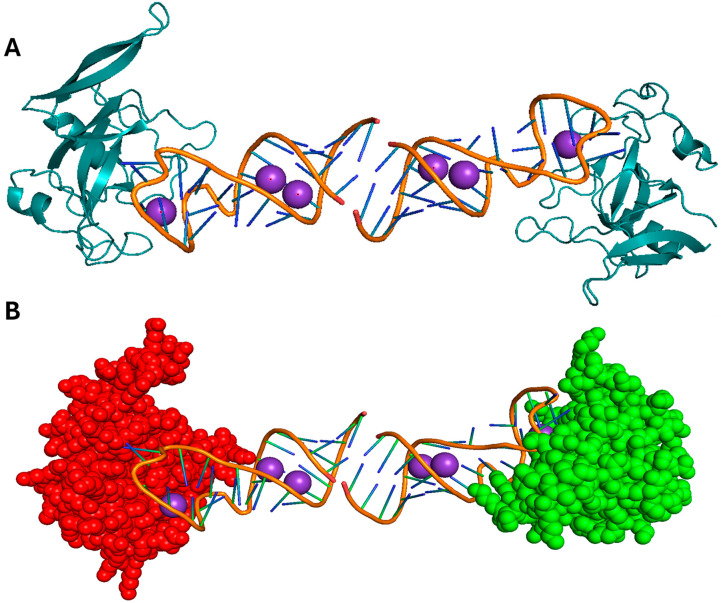
Structural analysis of restrictocin. (**A**) The crystal structure of restrictocin bound to DNA (PDB: 1JBS) as a cartoon, with the conserved α/β ribotoxin fold indicated. (**B**) Restrictocin visualized as spheres. All structures were visualized using PyMOL, version 2.5.8.

**Figure 9:**
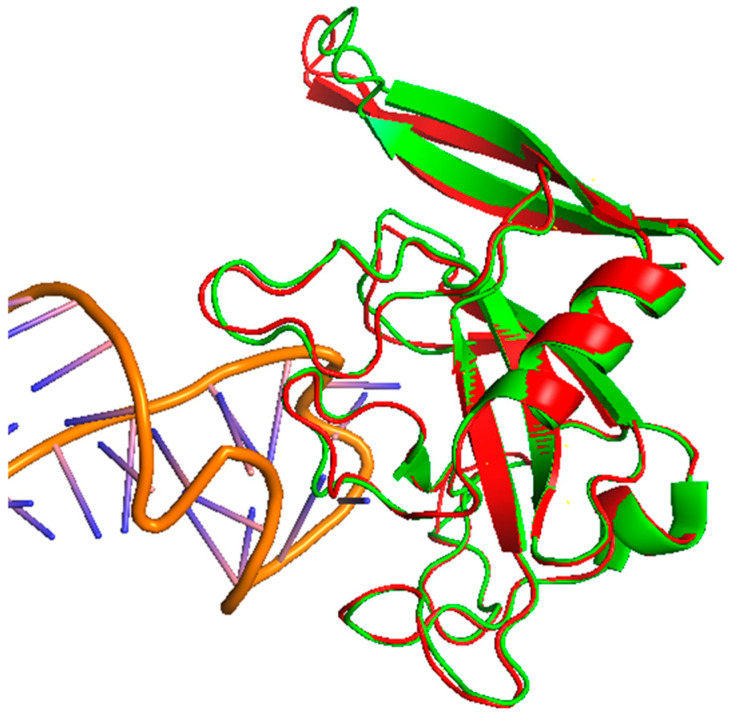
The predicted structure of Asp F1 using AlphaFold (green) aligned with the crystal structure of restrictocin (red, (PDB: 1JBS)), visualized in PyMOL, version 2.5.8.

Asp f1 is a ribotoxin secreted by *A. fumigatus* that cleaves rRNA and can be a potent allergen in allergic bronchopulmonary aspergillosis (ABPA) [57]. Asp f1 induces TLR2 and TLR4, which results in a Th2-skewed immune responses, eosinophilia, and chronic airway inflammation (Figure 6, [58]). Despite the unknown three-dimensional conformation of Asp f1, bioinformatic analyses and sequence alignment investigations indicate that it exhibits over 60% sequence homology with restrictocin, an extensively characterized ribotoxin derived from *Aspergillus restrictus* (Figure 8, [54]). These homologous proteins generally adopt a conserved α/β ribotoxin topology, which includes the formation of a pronounced active-site cleft that is stabilized by disulfide linkages, thereby implying that Asp f1 is likely to exhibit a comparable structural conformation and catalytic mechanism. We predicted the three-dimensional structure of the Asp f1 protein using AlphaFold and aligned it with the experimentally determined structure of restrictocin (Table 2, Figure 9). The predicted and reference structures exhibit a high degree of structural similarity.

### 3.3. Chitin and Chitin-Binding Proteins

**Figure 10:**
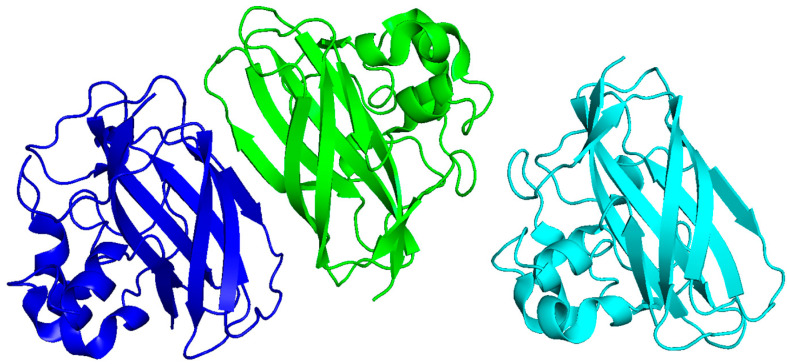
The crystal structure of the *S. marcescens* chitin-binding protein CBP21 (PDB: 2BEM), which is trimeric and contains conserved β -sandwich folds. Structures were visualized using PyMOL, version 2.5.8.

Chitin serves as a structural component of the fungal cell wall and functions as a pathogen-associated molecular pattern (PAMP). It is recognized by Dectin-1, NOD-like receptors, and TLR9, which leads to macrophage activation, IL-17 production, and, in certain instances, granulomatous inflammation (Figure 6). Binding to Dectin-1, a C-type lectin receptor, leads to receptor clustering and phosphorylation, which activates the NF-kB and MAPK pathways, leading to cytokine production and inflammation [59,60]. NOD-like receptors similarly can activate NF-kB [61]. Furthermore, fungi secrete chitin-binding proteins (CBPs) that stabilize newly synthesized cell walls and regulate chitin synthase and chitinase activity [62]. CBPs also defend against host immune responses, by sequestering the chitin and hiding it from immune detection [63]. While native chitin exhibits poor immunogenicity, chitin-binding proteins, like CBP21 from *Serratia marcescens*, a bacterial ortholog commonly used for fungal models, possess well-characterized structural features (Table 2, Figure 10). The trimeric form of CBP21 is stable and biologically relevant. CBPs exhibit conserved β-sandwich folds and surface residues that are important for interaction with chitin. The conserved β-sandwich folds also contribute to the stability of the structures that accommodate surface-exposed aromatic and polar residues, which enable selective interactions with chitin [64]. This structural configuration is important, since chitin is insoluble and needs specialized binding interfaces to promote enzymatic degradation [64,65]. Structural modelling of fungal CBPs suggests a parallel architecture [55].

### 3.4. Agglutinin-like Sequence Protein

**Figure 11:**
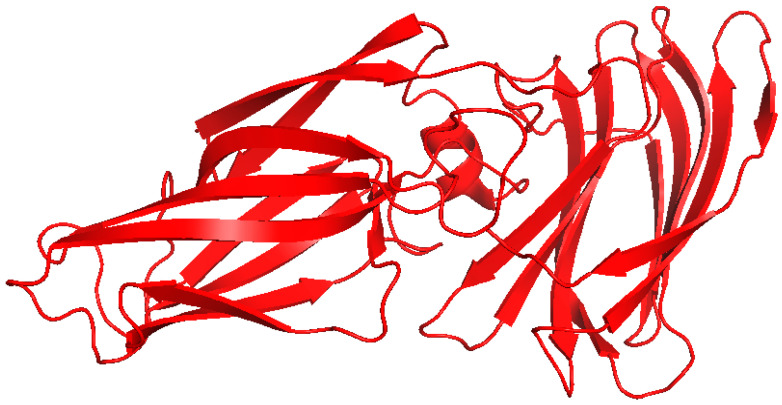
The crystal structure of Als3 from *C. albicans* (PDB: 4LE8). This structure contains a β-barrel fold characterized by a distinctive hydrophobic peptide-binding cleft. Structure was visualized using PyMOL, version 2.5.8.

Agglutinin-like sequence protein 3 (Als3) is a well-characterized fungal adhesin/invasin and is recognized as a virulence factor of *C. albicans*. Als3 interacts with E-cadherin receptors on epithelial cells and N-cadherin receptors on endothelial cells, which aids in fungal attachment and promotes endocytosis [56,66,67]. Epithelial cells that are exposed to *C. albicans* activate the NF-κB, MAPK, and PI3K signaling pathways, shortly after initial contact (Figure 6, [68]). A Swiss-Model was used to predict the structural conformation of Als3, which revealed that it contains an N-terminal adhesive domain that forms a cleft to accommodate host receptors [68]. The crystal structure of Als3 was also solved, and showed that it has a β-barrel fold characterized by a distinctive hydrophobic peptide-binding cleft, which facilitates interactions with host proteins, including E- and N-cadherin (Figure 11, [69]).

## 4. Biologics Targeting Viral and Fungal Inflammation

Biologic therapeutics, including antiviral peptides (AVPs), antifungal peptides (AFPs), and mAbs have emerged as pivotal components in the formulation of targeted interventions for chronic infections [70]. These biologics not only impede pathogen proliferation but also modulate the host immune response to mitigate subsequent inflammatory processes [71]. Chronic inflammation is distinguished by the continual activation of pro-inflammatory signaling cascades and overproduction of cytokines. Numerous pathogens utilize immune evasion mechanisms that enable their sustained presence within the host, which intensifies the inflammatory reaction [72,73,74]. Biologics have been engineered to dampen these common inflammatory pathways by neutralizing the pathogen directly or attenuating subsequent immune activation [75].

**Figure 12:**
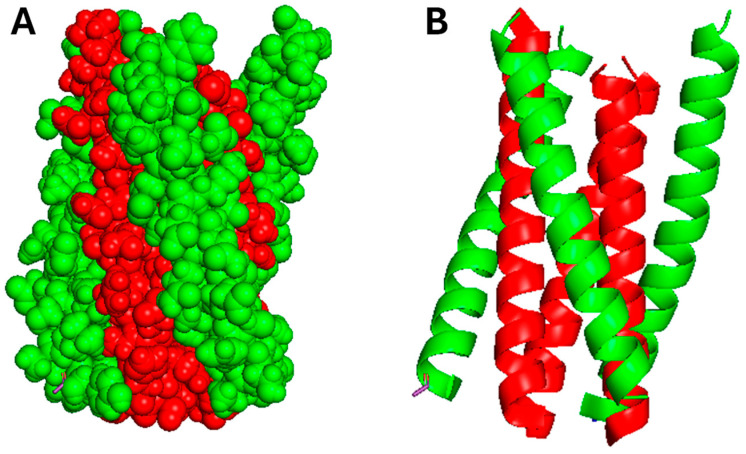
Crystal structure of N39, a trimeric peptide that mimics the NHR of gp41 (green) with T20 (red). (**A**) Spheres and (**B**) cartoon models, both visualized using PyMOL software, version 2.5.8.

AVPs are short peptide sequences that inhibit viral entry or replication while simultaneously providing immunomodulatory benefits. Enfuvirtide (T-20) is an FDA-approved peptide that obstructs the entry of HIV-1 by specifically targeting the gp41 envelope glycoprotein (Figure 12). gp41 itself is immunogenic, which leads to antibody production and the release of pro-inflammatory cytokines. Consequently, the inhibition of gp41 not only curtails viral replication but may also mitigates inflammation, including that which is linked to Tat-mediated cytokine induction. The crystal structure of gp41 in complex with T20 was obtained using a peptide (N39) that mimics the N-terminal heptad repeat region (NHR) of gp41, which inherently forms a coiled coil. T20 occupies the hydrophobic grooves of this NHR mimic, effectively obstructing the formation of the six-helix bundle, which is needed for membrane fusion [76,77]. Investigational peptides such as C34 and HIV-1 inhibitor virus-inhibitory peptide (VIRIP) were developed to inhibit HIV-1 fusion [78,79]. C34, which is derived from the heptad repeat 2 (HR2) domain of gp41, inhibits the formation of the six-helix bundle that is essential for viral fusion. Lipid-conjugated derivatives, including sifuvirtide and albuvirtide, went through the preliminary phases of clinical trials in China, and had favorable safety profiles, tolerability, and suppression of viral replication in patients with prior treatment failure [80,81,82]. Albuvirtide received regulatory approval in China in 2018 following phase III clinical trials that demonstrated sustained reductions in HIV-1 viral load when used in conjunction with standard antiretroviral therapy [83]. VIRIP (VIR-576) is a peptide fragment derived from α1-antitrypsin that directly binds to gp41 and obstructs its insertion into the membranes of host cells [84]. In a Phase I/IIa clinical trials with 18 subjects, VIR-576 was generally tolerated and had a favorable safety profile. The use of VIR-576 as a short-term monotherapy in treatment-naïve HIV-1 patients resulted in a substantial decrease in plasma viral load exceeding one order of magnitude, indicating both its tolerability and antiviral efficacy [85].

Peptide-based vaccines targeting E6 and E7 of HPV are designed to provoke cytotoxic T cell response to reduce inflammation and viral persistence. ISA101, a synthetic long-peptide vaccine that targets E6 and E7, induced HPV-specific CD4^+^ and CD8^+^ T cell responses in preclinical models [86]. A Phase II clinical trial examined the synergistic effects of ISA101 in combination with the programmed cell death protein 1 (PD-1) inhibitor nivolumab in 26 individuals diagnosed with advanced HPV-positive oropharyngeal carcinoma. Within this cohort, 3 patients had a partial response (PR), including two individuals who had not previously exhibited a response to immune checkpoint inhibitor therapy. Booster immunizations were delivered to nine patients, and the interval to response varied between 127 and 307 days. Adverse events associated with ISA101 were mostly minor, but autoimmune events were observed in 7.7% of the cohort. These results suggest that the combination of ISA101 and nivolumab has a benefit in patients who have failed therapy. This study highlights the potential efficacy of peptide-based immunotherapeutic strategies in malignancies driven by viral pathogens [87].

Additional peptides currently under development target inflammatory viral proteins such as HBx and LMP1, to disrupt their interactions with host signaling proteins that activate NF-κB [86,88]. A TRAF6-inhibitory peptide was identified from a bead-based AlphaScreen assay that interferes with the interaction between LMP1 and TRAF6 [89]. This peptide could be used to reduce NF-κB activation in vivo. Synthetic peptides derived from the C-terminal domain of LMP1 have also been synthesized, which mimic the signaling domains of LMP1. These peptides function as decoys by competitively binding to adaptor proteins such as TRAF2 and TRADD, obstructing LMP1 interaction. This sequestration impedes NF-κB signaling and triggers apoptosis in EBV-transformed cells [90].

AFPs also have dual functionalities by targeting fungal membranes while concurrently attenuating host inflammatory responses [91]. Naturally occurring peptides, such as Histatin 5 from human saliva, exhibit antifungal activity against *C. albicans* and contribute to the limitation of fungal-induced epithelial inflammation [92]. LL-37, a cathelicidin, has antifungal and anti-inflammatory properties by inhibiting the release of pro-inflammatory cytokines [93]. At present, LL-37 is undergoing both preclinical and clinical development. Phase I/II clinical investigations involving the management of venous leg ulcers revealed that the administration of low-dose topical formulations significantly enhances wound healing [94]. Human defensins (hBD-1, hBD-2) also disrupt fungal membranes and alter innate immune pathways, including TLR signaling and macrophage activation [95]. The use of β-defensins, specifically hBD-2, are in preclinical development and have demonstrated encouraging anti-inflammatory and antifungal properties across various animal models. Although clinical trials have not yet commenced, the observed efficacy and in vivo safety indicate potential for further development [96]. NP339, a synthetic AFP, is presently under investigation for its therapeutic potential in treating candidiasis while minimizing immune overactivation [97]. NP339 exhibits rapid fungicidal activity against various fungal pathogens and does not promote resistance development. In addition, NP339 was effective in animal models and Phase I clinical trials will soon begin [98,99].

mAbs are effective in the regulation of inflammatory responses that are associated with chronic infections [100]. Infliximab and Adalimumab are anti-TNFα antibodies and mitigate the excessive signaling of TNFα. Excessive TNFα signaling is frequently elevated in the presence of viral proteins, and during fungal inflammation mediated through Dectin-1 or TLR signaling pathways [101,102]. Tocilizumab, an antibody that specifically targets the IL-6 receptor, was approved for autoimmune disorders and conditions characterized by cytokine storms and was recently investigated for use with severe viral infections. During the COVID-19 pandemic, large clinical trials demonstrated that tocilizumab decreased mortality rates and enhanced recovery outcomes in patients hospitalized with severe disease. Despite its lack of widespread endorsement for other viral infections, its efficacy in the context of COVID-19 demonstrates its utility in mitigating cytokine-mediated complications associated with viral diseases (Table 3, [103]). The use of tocilizumab for treatment of fungal diseases, including ABPA, is currently under exploration due to the known role of IL-6 in exacerbating the inflammatory responses. Initial research indicates that the inhibition of IL-6 signaling may alleviate pulmonary inflammation and enhance respiratory function in patients afflicted with ABPA. More stringent clinical trials are required to evaluate the safety profile, optimal dosing parameters, and overall efficacy in relation to fungal-associated immune dysregulation [104,105]. Anakinra, which acts as an IL-1 receptor antagonist, is being assessed for its capacity to inhibit IL-1β–mediated inflammation that occurs in response to fungal inflammasome activators and viral proteins that interact with innate immune sensors (Table 3 [106,107]). In preclinical models, anakinra was effective in diminishing IL-1β production and mitigating inflammation in patients with cystic fibrosis and chronic granulomatous disease, which have an increased risk for fungal infections and the activation of inflammasomes [108]. Despite compelling pre-clinical evidence, there are no clinical trials examining the use of anakinra for fungal infections. Most of the research focuses on broader inflammatory disorders or autoimmune diseases. Considering the encouraging preclinical findings, further clinical investigations to evaluate anakinra for the treatment of fungal infections characterized by inflammation should be performed [109].

In addition to biologic agents that directly target pathogens, there is an increasing focus on therapies that target the common molecular mechanisms of inflammation that are initiated by both viral and fungal infections. A pivotal pathway in this context is the activation of NF-κB, which is exploited by viral proteins and is activated through fungal PRR signaling. Bortezomib, a proteasome inhibitor that has been approved for the treatment of multiple myeloma, inhibits the degradation of IκB and consequently prevents the activation of NF-κB (Table 3). Bortezomib has anti-inflammatory effects in experimental models of both viral- and fungal-induced inflammation [110]. Likewise, dimethyl fumarate, which was originally approved for the management of multiple sclerosis, inhibits NF-κB activity and demonstrated potential in treating neuroinflammation linked to HIV [111]. Colchicine, a microtubule inhibitor that is utilized in the treatment of gout, obstructs inflammasome assembly and reduces IL-1β levels in inflammation driven by infections. Given the impact of heightened IL-1β signaling in the context of fungal infections, there is interest in the synergistic application of antifungal agents alongside targeted immunomodulatory therapies [112]. The use of colchicine or alternative inflammasome-inhibiting treatments, such as anakinra, alongside traditional antifungal regimens can become a host-directed therapeutic approach that reduces both fungal load and immune-mediated damage to tissues.

## 5. Limitations of Current Biologics and the Potential of Conjugated Therapeutic Proteins

Despite the potential of biologics, including mAbs, AVPs, and AFPs, in the management of chronic inflammation associated with viral and fungal infections, numerous challenges impede their sustained efficacy and clinical applicability. A principal limitation is their abbreviated plasma half-life, particularly pertinent to peptides, which frequently necessitate repeated administration or formulation modifications to sustain therapeutic concentrations [113]. Furthermore, restricted tissue penetration, particularly into immune-privileged regions such as the central nervous system or deeply embedded fungal reservoirs, constrains the accessibility of many antibodies and peptides [114]. Another significant concern is the potential for immunogenicity, where recurrent administration may elicit host immune responses against the therapeutic agent itself, which diminishes efficacy and possibly induces adverse reactions [115]. Biologics frequently exhibit a lack of specificity for the inflammatory microenvironment, operating systemically rather than accurately targeting inflamed or infected tissues. This phenomenon may result in off-target effects, including generalized immune suppression, which could render patients susceptible to secondary infections or hinder the clearance of the primary pathogen [116]. Finally, in the context of AFPs and AVPs, proteolytic degradation by host enzymes can further diminish bioavailability, necessitating chemical modifications or protective delivery methodologies [117].

To address these challenges, there is an increasing interest in engineered conjugated therapeutic proteins, which combine biologic molecules with targeting, stabilizing, or effector domains to enhance functional capacity, selectivity, and pharmacokinetics. For example, AVPs or AFPs can be conjugated to antibody scaffolds or Fc fragments to augment stability and improve tissue distribution. Such conjugates preserve antimicrobial activity while increasing the structural stability and specificity inherent to antibodies. Likewise, mAbs may be conjugated to cytokine inhibitors, signaling inhibitors, or enzymatic payloads, resulting in multifunctional therapeutics capable of neutralizing a pathogen and modulating the local immune response [118]. Recent preclinical investigations have evaluated the viability of such constructs within inflammatory and oncological contexts. For example, antibody–drug conjugates that specifically target immune cells were designed to deliver anti-inflammatory agents, such as PDE4 inhibitors or glucocorticoids, which resulted in a diminished release of cytokines in human monocytes and peripheral blood mononuclear cells [119,120]. Additionally, bifunctional constructs that integrate antigen targeting with both immunomodulatory cytokines and cytotoxic agents have also exhibited therapeutic potential in murine experimental models [120,121]. Although these multifunctional mAbs have yet to be utilized in clinical practice for infectious diseases, their success with other disease states implies promise for host-directed therapeutic strategies for inflammation induced by pathogens.

Conjugated proteins present an opportunity to administer anti-inflammatory payloads directly to inflamed tissues, such as by targeting NF-κB–active environments or cells expressing PRRs like Dectin-1 or TLRs. For example, a conjugated antibody that targets fungal β-glucan could facilitate the localized delivery of an inflammasome inhibitor, which would attenuate IL-1β secretion specifically at the infection site [118]. For viral infections, conjugates that include Tat- or HBx-targeting antibodies with cell-penetrating peptides could locally inhibit NF-κB activation without inducing systemic toxicity [122]. Delivery platforms, which include nanoparticles or liposomes, affixed with AVPs, AFPs, or mAbs, can be engineered to respond to inflammatory stimuli, such as pH variations or reactive oxygen species levels, which would release their therapeutic cargo exclusively within diseased tissues [122]. This intelligent targeting paradigm mitigates collateral effects and enhances therapeutic efficacy. Recent advancements in protein engineering and chemical conjugation methodologies now facilitate site-specific, stable, and scalable production of such biologics, which could lead to a new generation of therapeutics [123]. These advancements include the implementation of engineered antibody scaffolds, and novel conjugation chemistries, such as thiol-selective modification and enzymatic click reactions that facilitate the controlled and reproducible attachment of therapeutic payloads [121,124]. Such site-specific methodologies reduce heterogeneity, augment pharmacokinetics, and enhance safety by mitigating off-target effects [125]. Collectively, these innovations establish a robust technological framework for the translation of conjugated proteins from laboratory to clinical application.

## 6. Challenges and Future Directions

Before engineered conjugated proteins can be used to treat chronic inflammation resulting from persistent infections, numerous challenges must be overcome to fully achieve their clinical efficacy [126]. A major hurdle pertains to the effective delivery of these conjugates to chronically inflamed tissues, including mucosal surfaces, pulmonary regions, and other affected organs. Such sites frequently exhibit complex biological barriers, including thick mucus layers, altered vascular permeability, fibrotic extracellular matrices, and infiltration by immune cells, all of which can impede the penetration, retention, and bioavailability of large biologics (Table 4, [127]). The development of advanced delivery systems, such as nanoparticles, liposomes, inhalable formulations, or ligands specific to target tissues, is imperative to overcome these physical and biochemical barriers to ensure that therapeutic concentrations effectively reach the sites of inflammation [128]. The issues of immunogenicity and rapid degradation persist as significant concerns for protein therapeutics. Engineered conjugates, particularly those incorporating non-human sequences or innovative linkers, are susceptible to eliciting immune responses that may neutralize the therapeutic agent or promote adverse effects. Additionally, proteases that are prevalent in inflamed tissues and systemic circulation can rapidly degrade peptides and proteins, resulting in reduced half-life and diminished efficacy.

Addressing these challenges necessitates the application of sophisticated protein engineering techniques, such as amino acid substitutions aimed at diminishing immunogenic epitopes, chemical modifications (e.g., PEGylation or glycosylation), the cyclization of peptides to enhance stability, and the incorporation of D-amino acids or non-natural residues to confer resistance to proteolysis (Table 4, [129,130]). Furthermore, the production of stable, homogeneous conjugated proteins that meet clinical-grade quality standards and can be generated in sufficient quantities requires robust expression systems and purification protocols. The inherent complexity of fusion proteins, in the case of conjugated proteins, exacerbates the risks of aggregation, misfolding, and variability between batches, thereby complicating regulatory approval processes and commercial production. The potential for off-target effects and toxicity must also be rigorously assessed. While the targeting of inflamed tissues or pathogen proteins enhances specificity, unintended interactions with host proteins or disruption of the normal microbiota may lead to adverse consequences. Comprehensive preclinical safety evaluations and sophisticated targeting strategies are essential to mitigate such risks. Emerging drug resistance presents a significant obstacle in contemporary biomedical research. Pathogens possess the ability to mutate, modify surface proteins that are the target of antibody domains, or alter their membrane composition to avoid interaction with antimicrobial peptides. This reality underscores the necessity for continual surveillance and may necessitate the development of adaptable or multi-epitope targeting conjugates. The incorporation of AI-driven structural prediction tools such as AlphaFold provides promising avenues to expedite the design process; however, computational models are inherently constrained, particularly in terms of predicting the dynamics of flexible linkers, post-translational modifications, and interactions within complex physiological settings [131]. Experimental validation is indispensable, and the implementation of hybrid computational-experimental workflows will be necessary [132]. Despite these obstacles, significant progress in protein engineering, delivery technologies, and computational modelling is occurring at an accelerated pace. The field is on the brink of producing increasingly sophisticated conjugated proteins that can selectively target pathogens and modulate chronic inflammation, thereby offering potential solutions for novel therapies against persistent infectious diseases [133].

## Figures and Tables

**Table 1:** Summary of viral proteins that induce inflammation.

Viral Protein	Virus	Mechanism of Inflammation	Structural Features	Known Motifs or Binding Sites	PDB	Refs.
Tat	HIV-1	NF-κB activation; cytokine induction (IL-6, TNF-α, IL-1β)	Intrinsically disordered with transient helices	Interacts with cyclin T1 via core region (aa 49–57)	1JFW	[22]
HBx	HBV	NF-κB activation via IKK complex; oxidative stress; cytokine upregulation	Multiple helical segments and a flexible C-terminal region	Interacts with IKKγ through C-terminal motifs	None ^1^	[23,24]
E6	HPV	Degrades p53, activates NF-κB and promotes cytokine expression	Two zinc-binding domains	Binds E6AP via two LxxLL motifs (aa 95–99)	4GIZ	[25]
E7	HPV	Inactivation of Rb; cell cycle dysregulation; NF-κB pathway stimulation	Zinc-binding C-terminal domain	Zinc-binding motif CxxC (aa 49 to 52)	2EWL	[26]
LMP1	EBV	Mimics TNFR; constitutive NF-κB activation; cytokine storm (IL-6, IL-8)	Predicted α-helical domains; partially disordered	C-terminal activation regions (CTAR1 and CTAR2) mediate TRAF binding	None ^1^	[27]

^1^ For structures with no PDB structure, predictions were made using AlphaFold. aa, amino acids.

**Table 3:** Summary table of biologics used to target common infection-induced inflammatory pathways.

Targeted Mechanism	Shared in	Therapeutic Biologics	Mechanism of Action
NF-κB pathway	Viruses, Fungi	Bortezomib, Anti-TNF mAbs	Inhibits IκB degradation, blocks TNF-driven activation
IL-6/IL-1 cytokine axis	Viruses, Fungi	Tocilizumab, Anakinra	IL-6R/IL-1R blockade to reduce cytokine storm

**Table 4:** The major hurdles and possible solutions for the use of therapeutic proteins.

Major Challenge	Description	Proposed Solutions & Strategies	Refs.
Targeted Delivery & Bioavailability	Complex biological barriers at inflamed sites, e.g., thick mucus or altered vasculatures, impede the penetration and retention of large protein conjugates.	Development of advanced delivery systems: Nanoparticles Liposomes Inhalable formulations Ligands specific to targeted tissues	[110,111]
Immunogenicity & Rapid Degradation	Conjugates with non-human sequences or novel linkers can trigger immune responses that neutralize the drug. High protease activity in inflamed tissues leads to rapid degradation and reduced half-life.	Protein Engineering Techniques: Amino acid substitutions to reduce immunogenicity Chemical modifications Peptide cyclization Incorporation of D-amino acids to resist proteolysis	[112,113]
Limitations of Computational Design	AI tools (e.g., AlphaFold) have constraints in predicting the behavior of flexible linkers, post-translational modifications, and complex physiological interactions.	Hybrid workflows that integrate computational design with rigorous experimental validation Iterative design-test-learn cycles to refine models	[114,115]

## Data Availability

Data is contained within the article.

## Abbreviations

The following abbreviations are used in this manuscript:

ABPA	Allergic Bronchopulmonary Aspergillosis
ADC	Antibody–Drug Conjugate
AFP	Antifungal Peptide
AVP	Antiviral Peptide
CBP	Chitin-Binding Protein
CTAR	C-Terminal Activation Region (e.g., in LMP1)
DAMP	Damage-Associated Molecular Pattern
EBV	Epstein–Barr Virus
ECM	Extracellular Matrix
FDA	Food and Drug Administration (USA)
HBV	Hepatitis B Virus
HBx	Hepatitis B virus X protein
HCV	Hepatitis C Virus
HIV	Human Immunodeficiency Virus
HPV	Human Papillomavirus
IKK	IκB Kinase
IL	Interleukin (e.g., IL-1β, IL-6)
LMP1	Latent Membrane Protein 1 (EBV)
LPS	Lipopolysaccharide
mAb	Monoclonal Antibody
NF-κB	Nuclear Factor Kappa-Light-Chain-Enhancer of Activated B Cells
NLRP3	NOD-, LRR- and Pyrin Domain-Containing Protein 3 (inflammasome)
PAMP	Pathogen-Associated Molecular Pattern
PDB	Protein Data Bank
PRR	Pattern Recognition Receptor
Rb	Retinoblastoma protein
Sap	Secreted Aspartyl Protease
Tat	Trans-Activator of Transcription (HIV-1)
TLR	Toll-Like Receptor
TNF-α	Tumor Necrosis Factor Alpha
TNFR	Tumor Necrosis Factor Receptor
TRAF	TNF Receptor-Associated Factor
TRADD	TNFR1-Associated Death Domain Protein
VIRIP	Virus-Inhibitory Peptide
